# Triggering of Apoptosis in Osteosarcoma 143B Cell Line by Carbon Quantum Dots via the Mitochondrial Apoptotic Signal Pathway

**DOI:** 10.1155/2020/2846297

**Published:** 2020-07-10

**Authors:** Yang Jiao, Yimin Guo, Yingcong Fan, Rui Wang, Xiang Li, Hao Wu, Zhichao Meng, Xin Yang, Yunpeng Cui, Heng Liu, Liping Pan, Talatibaike Maimaitijuma, Jiazhen Zhang, Yahong Wang, Yongping Cao, Tao Zhang

**Affiliations:** ^1^Department of Orthopedic Surgery, Peking University First Hospital, Beijing 100034, China; ^2^School of Materials Science and Engineering, Beihang University, Beijing 100191, China; ^3^Peking University Cancer Hospital & Institute, Beijing 100142, China; ^4^School of Basic Medical Sciences, Capital Medical University, Beijing 100069, China; ^5^Harbin Chengcheng Institute for Material and Life, Harbin 150500, China; ^6^School of Materials Science and Engineering, Zhengzhou University, Zhengzhou 450001, China

## Abstract

**Objectives:**

Carbon-based nanomaterials have gained attention in the field of biomedicine in recent years, especially for the treatment of complicated diseases such as cancer. Here, we report a novel carbon-based nanomaterial, named carbon quantum dots (CQDs), which has potential for cancer therapy. We performed a systematic study on the effects of CQDs on the osteosarcoma 143B cell line in vitro and in vivo.

**Methods:**

Cell counting assay, the neutral red assay, lactic dehydrogenase assay, and fluorescein isothiocyanate (FITC) Annexin V/Propidium iodide (PI) were used to detect the cytotoxicity and apoptosis of CQDs on the 143B cell line. Intracellular reactive oxygen species (ROS) were detected by the oxidation-sensitive fluorescent probe 2′,7′-dichlorofluorescein diacetate. The JC-10 assay was used to detect the mitochondrial membrane potential (MMP) of 143B cells incubated with CQDs. The effects of CQDs on the 143B cell line were evaluated by Western blot and immunofluorescence analysis of apoptosis-related proteins Bax, Bcl-2, cytochrome-C, caspase-3, cleaved-caspase-3, PARP1, and cleaved-PARP1. Male tumor-bearing BALB/c nude mice were used to investigate the antitumor effects of CQDs, and the biosafety of CQDs in vivo was tested in male BALB/c mice by measuring weight changes, hematology tests, and histological analyses of major organs.

**Results:**

CQDs exhibited a high cytotoxicity and induced apoptosis toward the 143B cell line. CQDs can also significantly increase the intracellular level of ROS and lower the mitochondrial membrane potential levels of 143B cells. CQDs increase apoptotic protein expression to induce apoptosis of 143B cells by triggering the mitochondrial apoptotic signaling pathway. The tumor volume in the CQD-treated mice was smaller than that in the control group, the tumor volume inhibition rate was 38.9%, and the inhibitory rate by tumor weight was 30.1%. All biosafety test indexes were within reference ranges, and neither necrosis nor inflammation was observed in major organs.

**Conclusions:**

CQDs induced cytotoxicity in the 143B cell line through the mitochondrial apoptotic signaling pathway. CQDs not only showed an antitumor effect but also high biocompatibility in vivo. As a new carbon-based nanomaterial, CQDs usage is a promising method for novel cancer treatments.

## 1. Introduction

Osteosarcoma is the most common primary malignant tumor in both children and adolescents and accounts for about 35% of malignant bone tumors [1]. In recent years, with the development of limb-salvaging surgery, neoadjuvant chemotherapy, immunotherapy, gene therapy, molecular targeted therapy, and other comprehensive treatments, the 5-year survival rate of osteosarcoma patients has increased to more than 80% [2]. However, such treatments have several deficiencies, such as cancer recurrence, incision infection, high expense, and side effects [3].

Carbon-based nanomaterials have been studied for biomedical applications in recent years. Compared with other nanomaterials, carbon nanomaterials have attracted attention due to their unique physicochemical and biological effects, such as low density, good chemical stability, low price, low toxicity, and strong cell penetration [4]. New forms of carbon nanostructures have been applied for drug delivery, antibacterial activity, clinical detection, antitumor activity, and bioengineering [5–19]. However, the biocompatibility and bioavailability of nanocarbon materials require further study [20–24]. To date, few studies have demonstrated that carbon-based nanomaterials present different cellular effects.

Here, we describe novel carbon-based nanomaterials, named, carbon quantum dots (CQDs), and their potential in cancer therapy. CQDs with a sheet-shaped structure are simply prepared via a unique pulse process, which covers the nanocarbon particles with hydroxyl groups. The average diameter of the carbon particles is about 5.58 nm, and the thickness of the nanosheets is equivalent to that of multilayer graphene. We then investigated the effects of colloidal CQDs on tumor cells in vitro to evaluate the CQDs' cytotoxicity, and a xenograft model was used to detect antitumor effects in vivo.

## 2. Materials and Methods

### 2.1. Preparation and Characterization of CQDs

**Figure 1:**
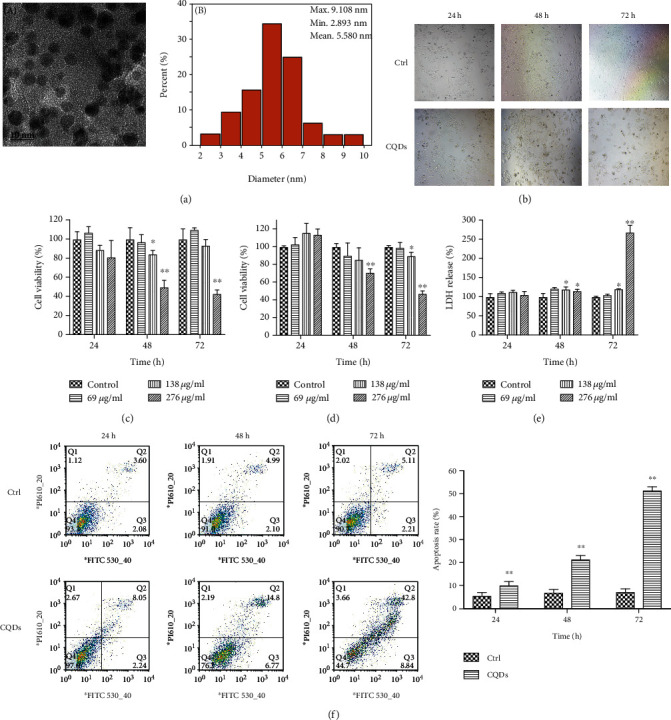
The basic characterization of the carbon quantum dots and the cytotoxicity and apoptosis of CQDs on 143B cell line. (a) TEM images of CQDs dispersed in a colloidal solution and size distribution of CQDs particles. (b) The morphology of 143B cells exposed to CQDs (276 *μ*g/ml) at 24, 48, and 72 h by optical microscopy (40x magnification). Cell viability of CQD-treated 143B cells after 24, 48, and 72 h determined by the (c) CCK-8 assay, (d) neutral red assay, and (e) LDH leakage assay. (f) Flow cytometric analysis of CQD-induced apoptosis in 143B cells using annexin V-FITC/PI and the columns show the apoptosis ratio of the cells. Results are mean ± SD of the triplicate experiments. Significant differences are marked with ^∗^ (^∗^*p* < 0.05; ^∗∗^*p* < 0.01 compared to the control).

Graphite plates (100 × 30 × 5 mm, 99.9% pure, JXSHA, Co., Ltd., China) were used as the source of carbon materials. The carbon quantum dots (CQDs) were prepared by liquid phase pulse electrolysis with high-temperature purified graphite plates as the carbon source. The high-temperature purification step is as follows: the graphite boat carrying the graphite plate to be purified is placed in a high-temperature furnace, and the temperature is raised to 2800°C under argon atmosphere, then the temperature is kept for 240 h, and finally, naturally cooled to room temperature. The purity of the purified graphite can reach 99.999%. The specific electrolysis method is place two high-temperature purified graphite plates in parallel in a water tank (150 × 80 × 60 mm), the plate spacing is 10 mm, then inject 500 ml of deionized water, and then connect the two graphite plates to the positive and negative poles of the unipolar pulse power supply (Jingxin Co., Ltd., China), and finally, a carbon quantum dot solution with a concentration of 1 mg/ml was prepared after electrolyzing (voltage = 5 V, frequency = 20 KHz) for 180 h. For transmission electron microscopy characterization, CQDs was cast onto a copper grid (300 mesh, Ted Pella Co., USA). After drying in air, the sample was observed using a JEM-2100F transmission electron microscope (FTEM, Tokyo, Japan) operating at 200 KV. The sample was dropped onto the silicon plate and dried in air. The primary particle size was measured using Image J software (National Institutes of Health, 9000 Rockville Pike, Bethesda, Maryland, America) across the diameter of the particles obtained by FTEM. A dynamic light scattering (DLS) spectrometer (DLS-7000, Otsuka Electronics Co., Inc., Osaka, Japan) was used to evaluate the size distribution of the samples.  Figure 1(a) shows the basic features of CQDs. We prepared 3 concentrations of CQDs (low concentration (69 *μ*g/ml), medium concentration (138 *μ*g/ml), and high concentration (276 *μ*g/ml)) for our study.

### 2.2. Cell Culture

The human osteosarcoma 143B cell line was purchased from KeyGEN BioTECH (Nanjing, China). Cells were cultured in Dulbecco's modified Eagle's medium (DMEM, Gibco, USA) with 10% fetal bovine serum (FBS, Gibco, USA) and 1% penicillin/streptomycin (PS, Gibco, USA) at 37°C in a 5% CO_2_ atmosphere.

### 2.3. Cell Counting Assay

The cell counting kit-8 (CCK-8, Dojindo, Japan) assay was used to evaluate cell viability. Cells were placed in 96-well plates at 5 × 10^3^ cells per well and incubated with CQDs for another 24, 48, and 72 h. Then, cells were twice washed with phosphate-buffered saline (PBS, Gibco, USA). CCK-8 working solution was added to each well and maintained for another 2 h at 37°C in a 5% CO_2_ atmosphere. The absorbance was recorded at 450 nm using a multifunction microplate reader (SYNGENE, Cambridge, UK).

### 2.4. Neutral Red Assay

The neutral red assay (NR, Beyotime, Shanghai, China) was also used to detect cell viability. 143B cells were plated in 96-well plates at 5 × 10^3^ cells per well in 100 *μ*l of culture medium, and then, CQDs was added. After incubation with CQDs for 24, 48, and 72 h, the medium was replaced with 200 *μ*l culture medium with 20 *μ*l NR solution and incubated for another 2 h. Then, 200 *μ*l of cell lysis solution was added to extract the dye. The absorbance was recorded at 540 nm.

### 2.5. Lactic Dehydrogenase Leakage Assay

A lactic dehydrogenase (LDH, Beyotime, Shanghai, China) leakage assay was used to determine the cell membrane integrity. Cells were planted in 96-well plates at a density of 5 × 10^3^ cells per well. After 24, 48, and 72 h exposure to CQDs, the supernatant was collected from individual wells and centrifuged. Supernatant (120 *μ*l) from individual cells was collected into another 96-well plate followed by addition of 60 *μ*l LDH assay reagent and incubation for 30 min. The absorbance was recorded at a wavelength of 490 nm.

### 2.6. Apoptosis Assay

The 143B cells were seeded and cultured in six-well plates. The cells were treated with high concentration (276 *μ*g/ml) of CQDs. Following treatment, the apoptosis ratio of the cells was quantified using the fluorescein isothiocyanate (FITC) Annexin V apoptosis detection kit I (Becton, Dickinson and Company, USA) at 24, 48, and 72 h. The cells were harvested and washed twice with cold phosphate-buffered saline (PBS, Gibco, USA) and then resuspended in 1X binding buffer at 1 × 106 cells/ml. Next, 100 *μ*l of the solution (1 × 105 cells) was transferred to a 5 ml culture tube. Then, 5 *μ*l of FITC Annexin V and 5 *μ*l propidium iodide (PI) were added. Cells were gently vortexed and incubated for 15 min at 25°C in the dark. Finally, 400 *μ*l of 1X binding buffer was added to each tube and analyzed by a flow cytometer (BD Biosciences, San Jose, CA, USA).

### 2.7. Assay of Intracellular Reactive Oxygen Species

Reactive oxygen species (ROS) were detected by the oxidation-sensitive fluorescent probe 2′,7′-dichlorofluorescein diacetate (DCFH-DA, Beyotime, Shanghai, China). 2′,7′-Dichlorofluorescein diacetate (DCFH-DA) passively diffuses into cells and is deacetylated by esterases to form nonfluorescent 2′,7′-dichlorofluorescein (DCFH). DCFH reacts with ROS to form the fluorescent product 2′,7′-dichlorofluorescein (DCF, green fluorescence) which can be measured as an indication of the amount of intracellular ROS [25]. 143B cells were seeded in a 12-well plate at a density of 1 × 10^5^ cells per well. After exposure to CQDs for 12, 24, and 48 h (The intracellular level of ROS changed earlier than apoptosis, so we advanced the detection time), cells were removed from the culture medium and washed three times with PBS. DCFH-DA diluted to a final concentration of 10 *μ*M with serum-free medium was added to cultures and incubated for 20 min at 37°C. The DCFH-DA diluent was then removed, and the cells were washed three times with serum-free medium. The fluorescence was read at 485 nm for excitation and 530 nm for emission with a multifunction microplate reader (SYNGENE, Cambridge, UK).

### 2.8. Mitochondrial Membrane Potential Assay

The JC-10 assay (Solarbio, Beijing, China) was used to detect the mitochondrial membrane potential (MMP) of 143B cells. JC-10 is a lipophilic, cyanocyanine cationic dye that selectively penetrates the mitochondria and can reversibly alter the emission of red fluorescence to green fluorescence in the case of reduced membrane potential (∆*Ψ*m). Healthy cells have a high membrane potential; in healthy cells, JC-10 selectively accumulates in the mitochondria and forms aggregates that show red fluorescence. In apoptotic cells, JC-10 localizes as a monomer exhibiting green fluorescence [26]. Cells were seeded in six-well plates at 3 × 10^5^ cells per well. CQDs (276 *μ*g/ml) were then added to the cells and incubated for 12, 24, and 48 h. Cells were incubated with JC-10 solution in buffer in the dark for 20 min. Then, cells were washed twice with buffer, and the fluorescence intensity (485 nm excitation and 530 nm emission for green fluorescent monomers, 525 nm excitation and 590 nm emission for red fluorescent aggregates) was recorded using the multifunction microplate reader, and cells were observed with a fluorescence microscope.

### 2.9. Western Blot Analysis

Western blotting was used to measure the expression of apoptosis-related proteins (Bax, Bcl-2, cytochrome-C, caspase-3, cleaved-caspase-3, PARP1, and cleaved-PARP1). After being incubated with CQDs (276 *μ*g/ml) for 72 h, cells were harvested, and the total protein was extracted in lysis buffer. The total protein was separated by 4–12% polyacrylamide gel electrophoresis precast glue (SurePAGE, Genscript, Nanjing, China) and transferred to polyvinylidene fluoride (PVDF) membranes (Millipore Corporation, USA). The membranes were blocked with 5% bovine serum albumin (BSA) in Tris-buffered saline-Tween (TBST) solution at room temperature for 1 h, and then incubated with primary antibodies against Bcl-2, Bax, Cytochrome-C, caspase-3, PARP1, and cleaved-PARP1 (Abcam, USA) and against cleaved-caspase-3 (Cell Signaling Technology, USA) at 4°C overnight. Membranes were incubated with the secondary antibodies (LABLEAD, Beijing, China) at room temperature for 1 h. Protein expression was visualized using an enhanced chemiluminescence detection system (GBOX-CHEMI-XT4, SYNGENE, Cambridge, UK).

### 2.10. Immunofluorescence Assay

Fixed and permeabilized 143B cells were blocked with 1% BSA in PBS at room temperature for 1 h and then incubated with primary antibodies at 4°C overnight. The cells were incubated with the secondary antibody at room temperature for 1 h in the dark and observed using a confocal laser scanning microscope (OLYMPUS, FLUOVIEW, FV1000, Japan).

### 2.11. In Vivo Assays

Healthy male BALB/c mice (6–8 weeks, 18–20 g) and male BALB/c nude mice (4–6 weeks, 16–18 g) were purchased from Beijing Vital River Laboratory Animal Technology Co. (Beijing, China). Before starting the study, all mice were acclimatized for 1 week in the 12 h light/dark cycle conditions and a controlled temperature with food and water freely available. The experimental design and procedures were approved by the Institutional Ethical Committee for Animal Care and Use of Peking University First Hospital, People's Republic of China (the ethical code: J201808).

#### 2.11.1. Antitumor Effect

Tumor-bearing mice were established by subcutaneous injection of 1 × 10^7^/ml 143B cells in 200 *μ*l PBS into the right flank region of 20 male BALB/c nude mice. After 4 weeks, the average tumor volume was about 340 mm^3^. The mice were randomized into two groups (10 mice per group): the control group (PBS) and the CQDs group. The tumor-bearing mice were given either 0.2 ml PBS or high concentration (276 *μ*g/ml) of CQDs via gastric perfusion once daily for 4 weeks. Each group of mice was weighed once per week, and the amount of gastric perfusion was adjusted according to body weight. After 4 weeks of gastric perfusion, all tumor-bearing mice were sacrificed. The tumor volume was calculated using the following formula:

(1) $$\begin{array}{c} V\left({\text{mm}}^{3}\right)=\frac{L\left(\text{mm}\right)\times W{\left(\text{mm}\right)}^{2}}{2}, \end{array}$$

where *L* represents the longest diameter and *W* represents the shortest/widest diameter of the tumor.

The tumor-inhibition rate was calculated using the following formula:

(2) $$\begin{array}{c} \left(\frac{\left[V_{\text{control}}–V_{\text{treatment}}\right]}{V_{\text{control}}}\right)\times 100\%, \end{array}$$

where *V* is tumor volume.

The inhibitory rate by tumor weight was calculated using the following formula:

(3) $$\begin{array}{c} \left(\frac{\left[W_{\text{control}}–W_{\text{treatment}}\right]}{W_{\text{control}}}\right)\times 100\%, \end{array}$$

where *W* is tumor weight.

#### 2.11.2. Biosafety Evaluation

Sixty male BALB/c mice were divided randomly into two groups (1-month group and 3-month group; *n* = 30 per group), and each of the two groups was divided into three small groups (*n* = 10 per group): 69 *μ*g/ml CQDs group, 276 *μ*g/ml CQDs group, and control group. Animals in the control group (*n* = 10) were given 0.2 ml of physiological saline (NS) via gastric perfusion once daily for 4 or 12 weeks. Animals in the high concentration group (*n* = 10) and low concentration group (*n* = 10) were given 0.2 ml CQDs (high and low concentrations, respectively) via gastric perfusion once daily for 4 or 12 weeks. Each group of mice was weighed every 2 weeks. After 4 and 12 weeks of gastric perfusion, CQD-treated mice and untreated mice were sacrificed, and blood was collected for serum biochemistry assays and a complete blood panel test. Blood (50 *μ*l) was detected for the complete blood panel test: white blood cells (WBC), lymphocytes (LY), monocytes (MONO), neutrophils (NEUT), red blood cells (RBC), hemoglobin (HB), and platelets (PLT). The rest of the blood was centrifuged to obtain serum for biochemistry assays: alanine transaminase (ALT), aspartate transaminase (AST), alkaline phosphatase (ALP), albumin (ALB), total protein (TP), total bilirubin (TBIL), triglyceride (TG), creatinine (Cr), and blood urea nitrogen (BUN). After 12 weeks of gastric perfusion, tissue was harvested from each group. Examined tissues included the heart, liver, spleen, lung, kidney, and brain. Samples were fixed in 10% formalin for histopathological analysis. The organ samples were processed into paraffin, sliced into 5-mm-thick sections, and stained with hematoxylin and eosin (H&E). Images were obtained using a microscope (OLYMPUS, Japan).

### 2.12. Statistical Analysis

The data are expressed as the mean ± standard deviation (SD) of at least three independent experiments using GraphPad Prism 6.0 (GraphPad, San Diego, CA, USA) in cellaur experiments. Significant differences among groups were determined with one-way analysis of variance (ANOVA) using statistical software SPSS version 24.0 in animal experiments. Statistically significant values were defined as *p* < 0.05 based on a two-tailed Student's *t*-test.

## 3. Results

### 3.1. CQDs Inhibits the Proliferation of 143B Cells and Increases the Rate of Apoptosis

After exposure to CQDs for 24, 48, and 72 h, CQDs inhibited the proliferation of 143B cells at 72 h by optical microscopy (Figure 1(b)). In order to confirm the inhibition of proliferation of 143B cells, a CCK-8 assay (Figure 1(c)), NR assay (Figure 1(d)), and an LDH leakage assay (Figure 1(e)) was performed. After 72 h of exposure to low concentrations of CQDs (69 and 138 *μ*g/ml), the viability of 143B cells was not significantly altered; however, cells exposed to the higher concentration of CQDs (276 *μ*g/ml) showed a significant decrease in proliferation (Figures 1(c)–1(e)).

Next, we assessed the rate of apoptosis using Annexin V-FITC flow cytometry following 24, 48, and 72 h of high concentration (276 *μ*g/ml) CQDs treatment. The apoptosis rate of 143B cells increased after treatment with high concentration (276 *μ*g/ml) CQDs (Figure 1(f)). These results indicate that CQDs decreased cellular viability and increased the apoptosis rate.

### 3.2. CQDs Activates the ROS-Mediated Mitochondrial Pathway

**Figure 2:**
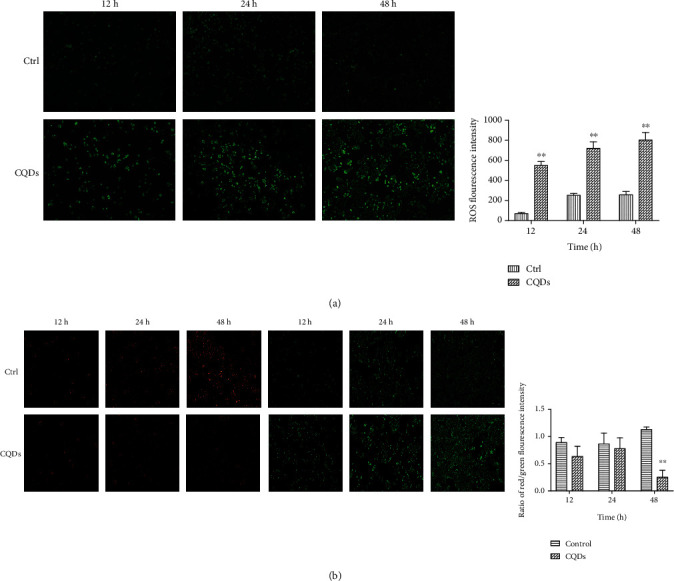
The ROS and MMP of 143B cells with and without CQDs treatment. (a) Images showing the intracellular levels of ROS in 143B cells. (b) Images showing the change of mitochondrial membrane potential in 143B cells. Significant differences are marked with ^∗∗^ (*p* < 0.01 compared to the control). Images were captured at 40x magnification.

As shown in Figure 2(a), high concentrations of CQDs (276 *μ*g/ml) significantly increased the intracellular level of ROS. A JC-10 assay was used to examine the mitochondrial membrane potential in 143B cells with and without CQDs treatment. As shown in Figure 2(b), exposure to high concentrations of CQDs significantly lowered the MMP levels of 143B cells at 12, 24, and 48 h compared to the control group.

### 3.3. CQDs Induces Apoptosis in 143B Cells via the Mitochondrial Apoptotic Signaling Pathway

**Figure 3:**
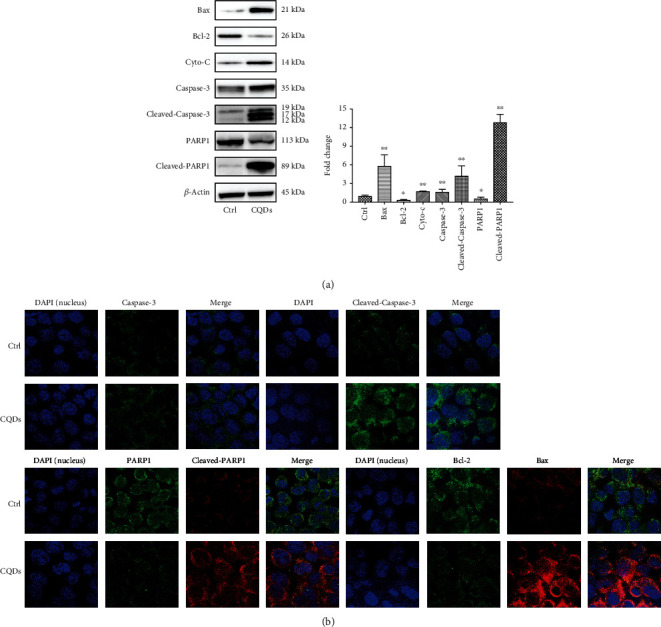
Expression levels of apoptotic proteins in 143B cells. (a) Western blotting was used to measure the expression of apoptosis-related proteins in 143B cells after exposure to CQDs. Results are mean ± SD of the triplicate experiments. Significant differences are marked with ^∗^ (^∗^*p* < 0.05; ^∗∗^*p* < 0.01 compared to the control). (b) Detection of apoptotic proteins in 143B cells by immunofluorescence assay and visualized by confocal laser scanning microscopy at 600x magnification.

The expression of protein Bcl-2 was significantly lower and Bax was significantly higher in 143B cell line treated with CQDs compared with the control group. The expression of PARP-1 in the CQDs group was higher than the control group. Caspase-3 was activated in cells treated with CQDs and resulted in the cleaved-caspase-3. The expression of cleaved-caspase-3 in CQDs group was higher compared to the control group. The expression of PARP1 was lower and cleaved-PARP1 was significantly higher in 143B cell line treated with CQDs compared with the control group (Figures 3(a) and 3(b)). The above data indicate that CQDs induces apoptosis of 143B cells via the mitochondrial apoptotic signaling pathway.

### 3.4. CQDs Has an Antitumor Effect and Good Biosafety In Vivo

**Figure 4:**
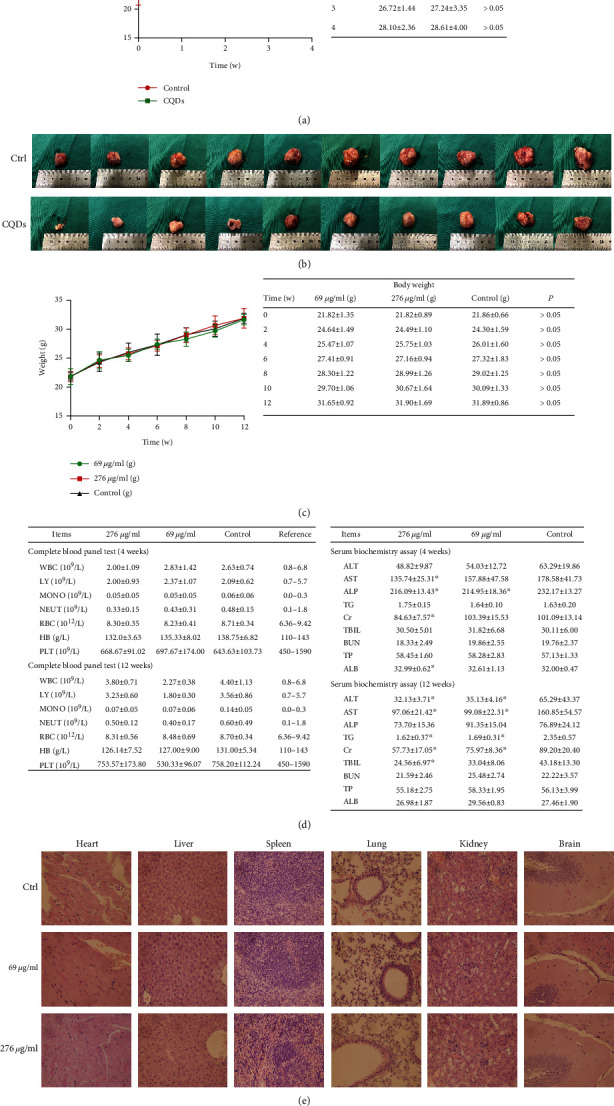
The antitumor effects and the biosafety of CQDs in vivo. (a) Body weight of tumor-bearing mice after treatment with CQDs or physiological saline for 4 weeks. (b) Image of the dissected subcutaneous tumors from the tumor-bearing mice receiving CQDs treatment. (c) BALB/c mice body weight after treatment with CQDs or physiological saline for 12 weeks. (d) Complete blood panel tests and blood chemistry analysis of BALB/c mice after treatment with CQDs for 4 and 12 weeks. (e) Images of H&E-stained organ samples from mice sacrificed at 12 weeks after gastric perfusion of CQDs. Images were captured at 400x magnification. Results are the mean ± SD of 10 samples. Significant differences are marked with ^∗^ (*p* < 0.05, compared to the control).

A murine xenograft model mimicking human osteosarcoma was established (Figure S1). The tumor volumes of the CQD (276 *μ*g/ml)-treated group and the control group before treatment were 346.7 ± 36.5 mm^3^ and 332.6 ± 55.2 mm^3^, respectively. After 4 weeks of treatment, all the tumor-bearing mice survived, and no significant body weight change was observed compared to the control groups (Figure 4(a)). The tumor volume in the CQD-treated group was smaller than that in the control group (2734.6 ± 282.0 mm^3^ and 4038.6 ± 342.6 mm^3^, respectively; Figure 4(b)). The tumor volume inhibition rate was 38.9%. The tumor weight of the CQD-treated group was 2.0 ± 1.1 g, which was lighter than the control group (2.9 ± 0.9 g). The inhibitory rate by tumor weight was 30.1%.

For biosafety of CQDs, after 4 and 12 weeks of treatment, all mice survived, and there was no significant body weight loss in any group (Figure 4(c)). Blood chemistry analysis was performed and complete blood panel tests were acquired after 4 and 12 weeks of CQDs treatment. All the test indices were within the reference ranges (Figure 4(d)). However, the ALT, AST, and Cr values were lower in the CQD-treated groups compared to the control group. Major organs were stained with H&E. None of the tested organs showed significant histological lesions after CQDs treatment (Figure 4(e)).

## 4. Discussion

In this study, we used the human osteosarcoma 143B cell line to investigate the potential cytotoxicity of new carbon-based nanomaterials (CQDs) in vitro and in vivo.

The CCK-8, NR assays, and LDH leakage assay indicated that cells exposed to CQDs had cytotoxicity in cell viability when the cells were exposed to a high concentration (276 *μ*g/ml) of CQDs. The results indicate a corresponding decrease in cell viability of 143B cells with CQDs.

Mitochondria are involved and play a central role in the integration and circulation of death signals initiating inside the cells (such as oxidative stress, DNA damage) in regulating cell death pathways. Cytotoxic agents such as radiation, nitrogen monoxide, arsenic, alloxan, streptozotocin, doxorubicin, mercury, and copper nanostructures induce apoptosis involving the mitochondria. Theses stimuli result in the formation of pores at mitochondrial membranes. Due to the formation of outer mitochondrial membrane (OMM) pores, the integrity of mitochondrial membranes gets disturbed, resulting in the decrease in the mitochondrial membrane potential (MMP) and release of two main groups of proapoptotic proteins, which normally become sequestered at intermembrane space into the cytosol. In this connection, overproduction of ROS causes oxidation of lipids, nucleic acids, and proteins, in a straightforward fashion, and as a consequence, loss of MMP as part of a positive response is developed [27].

The correlation between the increased ROS and decreased cell viability indicates that intracellular oxidative stress is associated with the cytotoxicity of CQDs. Increased ROS, an indicator of oxidative stress, is a common mechanism of cell damage caused by other carbon-based nanomaterials [28–30]. When ROS induces an antioxidant defense mechanism, oxidative stress can affect the cell integrity and induce cell protein inactivation, lipid peroxidation, mitochondrial dysfunction, and DNA damage and can ultimately be the main executor of apoptosis or necrosis [31–33]. Previous studies have suggested that carbon nanomaterials could induce intracellular oxidative stress by initiating an imbalance between the oxidant and antioxidant. Pristine graphene exhibited ROS-mediated toxic effects due to the interactions with the cell membrane [34]. Our results showed that exposure to CQDs induced a dose-dependent decrease of MMP in 143B cell line, indicating a decrease in functional mitochondria. It was determined that excess ROS production plays an important factor in mitochondrial damage, which disrupts the balance of intracellular ROS. Meanwhile, MMP played a key role in maintaining ROS homeostasis. The depletion of MMP induced by CQDs can increase the generation of ROS, leading to cytotoxicity. Similar effects were observed in treated cells among other types of carbon nanomaterials. Pristine graphene can induce cytotoxicity through the decrease of MMP and the increase of intracellular ROS, which then triggers apoptosis by activating the mitochondrial pathway. Mitochondria are the main source of ROS production and excess ROS induce apoptosis by destroying the MMP. The MMP of cells was studied to assess whether the CQD-induced ROS may be caused by impaired mitochondrial function. Exposure to CQDs decreased the levels of cellular MMP, indicating a reduction in functional mitochondria. Excess ROS production plays an important role in mitochondrial damage while MMP plays a key role in maintaining ROS homeostasis. In summary, CQD-induced depletion of MMP can increase ROS production, leading to cytotoxicity.

Mitochondrial malfunction affects various interlinked cellular pathways leading to the damage of intracellular components and unleash of cytochrome C. Following cytochrome C release, mitochondrial apoptotic pathway is activated resulting in apoptosis. The apoptosome initiates procaspase-9, an initiator caspase that is splitted into caspase-9. As a result, caspase-9 triggers executioner caspases for instance caspase-3, which helps in nuclear DNA split and seizing up of the cytoskeleton and nuclear lamina, forcing cells to presume an apoptotic, spherical structure. Mitochondrial membranes have Bcl-2 proteins, which stimulate or inhibit cell death via protein-protein interactions. Proapoptotic proteins, for instance Bax and Bad, help in allowing passage of the mPTP (mitochondrial permeability transition pore). Antiapoptotic proteins, for instance Bcl-2 and Bcl-xL, inhibit cell death by binding and hindering proapoptotic proteins. Caspase-3 is considered an important effector protease that is cleaved and activated during apoptosis [35]. Caspase-3 in turn cleaves a variety of cellular substrates, most notably PARP. PARP acts to help repair single-strand DNA nicks, and cleaved PARP is a useful marker of apoptosis [36]. High levels of radiation cause excessive activation of PARP-1, and this high level of PARP-1 can activate release of apoptosis-inducing factor (AIF). AIF is transferred from the cytoplasm to the nucleus and can promote DNA fragmentation and chromatin aggregation in the nucleus, resulting in apoptosis [37, 38].

Graphene can induce cytotoxicity by decreasing the MMP and increasing intracellular ROS and then trigger apoptosis by activating the mitochondrial apoptotic pathway [39, 40]. Another report states that carbon-based nanomaterials can induce mitochondrial damage through the mitochondrial apoptotic pathway [41]. We hypothesized that the main mechanism of CQD-induced osteosarcoma cell apoptosis was the activation of mitochondrial apoptosis-related proteins. The expression of protein Bcl-2 was significantly lower, and Bax was significantly higher in 143B cell line treated with CQDs compared with the control group. The expression of PARP-1 in the CQDs group was higher than the control group. Caspase-3 was activated in cells treated with CQDs and resulted in the cleaved-caspase-3. The expression of cleaved-caspase-3 in CQDs group was higher compared to the control group. The expression of PARP1 was significantly lower, and cleaved-PARP1 was significantly higher in 143B cell line treated with CQDs compared with the control group. These results show that CQDs can activate apoptotic proteins and induce apoptosis of osteosarcoma cells through the mitochondrial apoptotic pathway.

We also assessed the antitumor effect of CQDs in vivo and established a xenograft model by subcutaneous injection of 143B cells into the right flank region of BALB/c nude mice. After 4 weeks of CQDs treatment, all the tumor-bearing mice survived, and no significant body weight change was observed between the two groups. The tumor volume in the CQD-treated group was smaller than that in the control group, and the tumor volume inhibition rate was 38.9%. Our results are in agreement with other studies [42–49]. The tumor weight of the CQD-treatment group was less than in the control group, and the inhibitory rate by tumor weight was 30.1%, which is similar to studies of other nanomaterials [50, 51].

Next, we evaluated the biosafety of CQDs. After 12 weeks of treatment, all mice survived, and no significant body weight loss was observed. Blood chemistry analysis and complete blood panel tests were within reference ranges for healthy mice. These pieces of data coincide with the studies of other nanomaterials [52–54]. However, the ALT, AST, and Cr values were lower in CQDs groups compared to the control group. Whether CQDs protect the liver and renal function requires further research. Be, different from graphene and other nanomaterials, exhibited biological toxicity at high concentrations [52–55]. CQDs possess no obvious in vivo toxicity.

There are still some deficiencies in our study. For instance, we studied only one HOS cell line in vitro; we did not use animal tumor tissues to measure the expression of some key apoptotic proteins and did not explore the pharmacokinetics of CQDs in animals. We have not shown whether ROS scavenger could suppress CQD-induced ROS production and apoptosis. The tumor volume of tumor-bearing mice was decreased, but this phenomenon may be because CQDs play an important role in immunoregulation. Although mice brains were stained with H&E and showed no significant histological lesions, this result may be because CQDs cannot get through the blood-brain barrier. These important issues need to be investigated in our study in the future.

## 5. Conclusions

In conclusion, our present studies analyzed the cytotoxicity of CQDs with the osteosarcoma 143B cell line in vitro and its antitumor effect and biosafety in vivo. CQDs induced cytotoxicity in vitro through the mitochondrial apoptotic signaling pathway. Moreover, CQDs showed not only an antitumor effect but also high biosafety biocompatibility. As a new carbon-based nanomaterial, CQDs is a promising method for novel cancer treatments.

## Data Availability

All data generated or analysed during this study are available from the corresponding author on reasonable request.

## Additional Points

*Highlights*. (i) Carbon quantum dots have high biological effects as a new nanomaterial. (ii) Carbon quantum dots induced cytotoxicity in the 143B cell line through the mitochondrial apoptotic signaling pathway. (iii) Carbon quantum dots not only showed an antitumor effect but also high biosafety biocompatibility in vivo.

## Supplementary Materials

Supplementary Materials Figure S1: murine xenograft model. (a) Tumor-bearing mice were established by subcutaneous injection 143B cells into the right flank region of male BALB/c nude mice. (b) H&E staining of xenograft tumor slices. Images were captured at 400x magnification.
