# Supplementary material for: Triggering of Apoptosis in Osteosarcoma 143B Cell Line by Carbon Quantum Dots via the Mitochondrial Apoptotic Signal Pathway
Source: Biomed Res Int. 2020 Jul 10;2020:2846297. doi: 10.1155/2020/2846297 (PMC7369657; doi:10.1155/2020/2846297)
Supplement: Supplementary Materials — Figure S1: murine xenograft model. (a) Tumor-bearing mice were established by subcutaneous injection 143B cells into the right flank region of male BALB/c nude mice. (b) H&E staining of xenograft tumor slices. Images were captured at 400x magnification. [file 2846297.f1.pdf]

Supplementary material

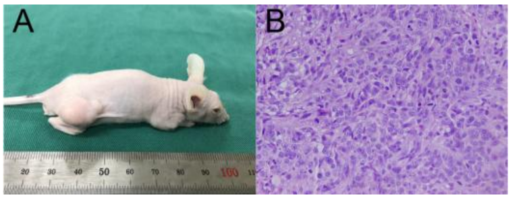

FIGURE S1. Murine xenograft model. (A) Tumor-bearing mice were established by subcutaneous injection 143B cells into the right flank region of male BALB/c nude mice. (B) H&E staining of xenograft tumor slices. Images were captured at 400x magnification.
